# Wear patterns of radular teeth in *Loligo vulgaris* (Cephalopoda; Mollusca) are related to their structure and mechanical properties

**DOI:** 10.1098/rsfs.2023.0082

**Published:** 2024-04-12

**Authors:** Svenja Hackethal, Ellen Schulz-Kornas, Stanislav N. Gorb, Wencke Krings

**Affiliations:** ^1^ Department of Cariology, Endodontology and Periodontology, Universität Leipzig, Liebigstraße 12, 04103 Leipzig, Germany; ^2^ Department of Mammalogy and Paleoanthropology, Leibniz Institute for the Analysis of Biodiversity Change, Martin-Luther-King-Platz 3, 20146 Hamburg, Germany; ^3^ Department of Electron Microscopy, Institute of Cell and Systems Biology of Animals, Universität Hamburg, Martin-Luther-King-Platz 3, 20146 Hamburg, Germany; ^4^ Department of Functional Morphology and Biomechanics, Zoological Institute, Christian-Albrechts-Universität zu Kiel, Am Botanischen Garten 1–9, 24118 Kiel, Germany

**Keywords:** feeding structures, mechanical properties, abrasion, structural failure, interface

## Abstract

Radular teeth have to cope with wear, when interacting with ingesta. In some molluscan taxa, wear-coping mechanisms, related to the incorporation of high contents of iron or silica, have been previously determined. For most species, particularly for those which possess radulae without such incorporations, wear-coping mechanisms are understudied. In the present study, we documented and characterized the wear on radular teeth in the model species *Loligo vulgaris* (Cephalopoda). By applying a range of methods, the elementary composition and mechanical properties of the teeth were described, to gain insight into mechanisms for coping with abrasion. It was found that the tooth regions that are prone to wear are harder and stiffer. Additionally, the surfaces interacting with the ingesta possessed a thin coating with high contents of silicon, probably reducing abrasion. The here presented data may serve as an example of systematic study of radular wear, in order to understand the relationship between the structure of radular teeth and their properties.

## 1. Introduction

Surfaces of teeth can have all kinds of structure-related functions, as, for example, reducing the wear when the surface interacts with abrasive food. In vertebrates, the wear patterns directly relate to the preferred ingesta (i.e. food, the substrate to which the food is attached, particles on the food, etc.) and dietary preferences [1–3]. In molluscs, the origins of wear in teeth are completely understudied. Only few studies have investigated the radular tooth wear systematically, but mostly anecdotally. The volume loss has been documented and characterized for docoglossan teeth of Polyplacophora and Patellogastropoda [4] and rhipidoglossan teeth of the neritid *Vittina turrita* [5]. Additionally, the quantities of teeth affected from wear have been examined and linked with the abrasiveness of the ingesta in Heterobranchia [6] and with the proposed function of different teeth in the rhipidoglossan radula [5]. In these latter studies [5,6], feeding experiments with abrasive sand papers have been performed. The wear in wild-living specimens has only been documented for Polyplacophora with the focus on tooth volume loss [4], but a more profound and detailed understanding of radular wear is lacking, especially with regard to wear-coping mechanisms.

**Figure 1.**
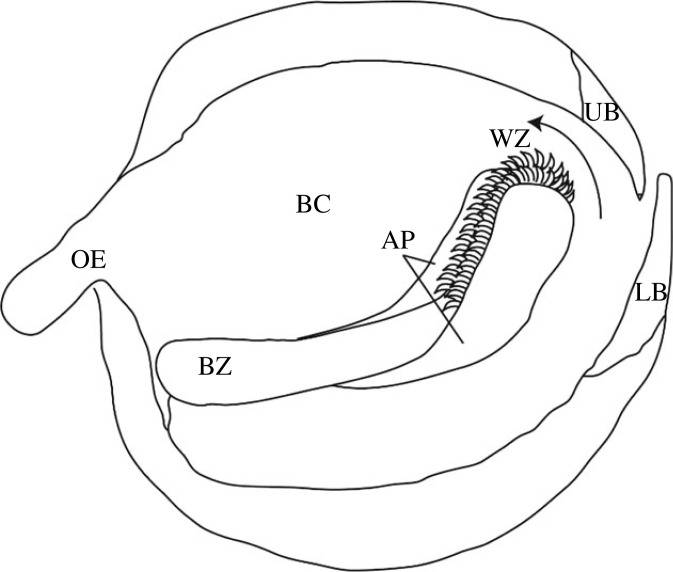
Schematic illustration of the cephalopod feeding structures, adapted and altered after [7]. The radula, consisting of a chitinous membrane with embedded teeth, is flanked to each side by alary processus, which bend the radula. This is bolstered by small roundish odontophoral cartilages. During feeding the radula is probably retracted into the buccal cavity to deliver food parts into the oesophagus. Abbreviations: AP, alary processus (= hyaline shield); BC, buccal cavity; BZ, building zone; LB, lower beak; OE, oesophagus; UB, upper beak; WZ, working zone.

The molluscan radula is an important autapomorphy of this phylum, showing a high diversity with regard to morphology, chemical and biomechanical properties. The structure consists of a chitinous membrane with rows of embedded teeth, which puncture prey, grab larger parts, gather small particles, or loosen algal covers from stones, depending on the species. They consist of densely packed chitin fibres, which spread from the membrane across the tooth basis, stylus and finally to the cusps. Depending on the molluscan taxa, membrane and teeth can be laterally flanked by two large chitinous alary processus, which bend the radula (figure 1). The radula can be, depending on the taxa, supported and bolstered by odontophoral cartilages of a variety of sizes and shapes. During feeding the radula is moved by various muscles, enabling coordinated and precise feeding motions.

Teeth and membrane are constantly secreted in the building zone or the in radular sac and become maturated during radular ontogeny before they enter the radular working zone, where teeth directly interact with the ingesta (i.e. food, particles on the food, substrate to which the food is attached, etc.). After some time, the used teeth are lost in the degenerative zone. Even though the radula is constantly renewed, mechanisms which reduce wear and structural failure are present and can be understood as wear coping mechanisms. In Polyplacophora [8–15], Patellogastropoda (e.g. [4,16–18]), paludomid [19–21], heterobranch [22] or nudibranch gastropods [23], this includes the incorporation of high proportions of iron (Fe), calcium (Ca) or silicon (Si) in the interacting surfaces, leading to harder tooth cusps, to deal with hard and abrasive ingesta, such as stone surfaces, Porifera spiculae, or Foraminifera. In Polyplacophora and Patellogastropoda, the tooth cusps of the dominant teeth are almost completely filled with such incorporations, whereas the tooth cusps of paludomid and nudibranch gastropods possess only a thin outer layer with high proportions of Ca or Si.

During feeding actions, such as piercing or scratching, high forces have to be transferred onto the hard target surfaces without high structural failure of the tooth. In radular teeth, this is enabled by the morphology (e.g. by providing broad and thick cusps during scratching action) or mechanical property gradients along the tooth, usually with the cusp or tip as the stiffest region and at the embedment in the membrane as the most flexible region (e.g. [11,17,18,22–40]). This increases strain at the basis, which reduces high stress values in the tooth tip when interacting with food, but also allows a bending or swerving, when the stress is too high. Besides, in some taxa, this bending enables an interlocking of teeth from adjacent tooth rows, which transmits forces from one tooth to another distributing the stress over several teeth [26,41,42] (termed ‘collective effect’ by [15,33,34]). Mechanical property gradients, enabling this biomechanical behaviour, can have their origin in (a) the morphology of the teeth (high aspect ratio), (b) the distributions of inorganic compounds (more minerals at the cusp) (e.g. [4,8–18]), (c) the distribution of proteins, the degree of tanning and cross-linking of the chitin [22,23,29,37,43–46], (d) the chitin fibre density [19], and (e) the water content of teeth and radular membrane [15,34,35]. Additionally, (f) the radular supporting structures (odontophoral cartilages, radular bolsters; e.g. [47–49]) seem to support the radular membrane and reduce stress concentration by acting as a cushion or muscular hydrostat [5,6,33,50–52].

The here presented work aims at describing and characterizing the knowledge gap of wear patterns on radular teeth in molluscs. The properties of the teeth were tested by a set of methodological approaches (scanning electron microscopy (SEM), confocal laser scanning microscopy (CLSM), nanoindentation, energy-dispersive X-ray spectroscopy (EDX/EDS)) to gain insight into their biomechanical and chemical characteristics. These were linked to the different wear patterns, and a hypothesis of the interaction with the ingesta and wear processes on mollusc tooth surfaces is developed.

As model species, we have chosen the cephalopod *Loligo vulgaris* Lamarck, 1798, because its radula contains per row only seven large teeth that have large worn-down areas, which can be measured by optical metrology approaches. This species has a broad food spectrum and feeds on fish, crustaceans, smaller squids and polychaets [53–60] and specimens in larger quantities are easily accessible. Cephalopods mainly cut and disable their prey with their chitinous beaks [53,61–63] (for reviews on cephalopod feeding, see [64–66]; for review on beaks, see [67]). The radula is rather used for the piercing of the prey, to clean crustacean carapaces and to transport it into the oesophagus [53,64,68–76]. Only in some cephalopods, such as *Octopus*, is the radula, together with teeth on the salivary papilla, involved in drilling of hard shells [77–84].

The cephalopod radula is supported by odontophoral cartilages and surrounded to the sides of the radular membrane by hyaline shields (= alary processus), which bend the radula and create a region (bending plane) where the teeth are erect [64,74,75,81,85–94] (for reviews on the anatomy of cephalopod mouth parts, see [7,95–97]). The tooth morphology has been previously well studied (e.g. [74,86,87,98,99]), also on fossil cephalopods (e.g. [100–102]). Wear on the cephalopod teeth was previously described [68,74,82,89,90,103–106], but not in a systematic manner and not in connection with mechanical properties, as there are no data on those. Except for *Nautilus*, which contained high concentrations of Ca and Si in its radula [107], the cephalopod radula seems to be rather chitinous and unmineralized [108–112], similar to beaks [113–116]. In one previous study on *L. vulgaris* radula, some small contents of inorganics (Mg, Ca, P and Cu) were detected [46], which indicates that a cross-linking through alkaline earth and transition metals could be present, similar to the situation in the radula of the vetigastropod *Megathura crenulata* [29] or cuticle [20,117–120].

We expect that wear can be found in the surfaces that have intimate interaction with ingesta and propose the following hypotheses. (a) The wear patterns differ between the tooth positions along the radula regions following the ontogeny and positioning within one tooth row from centric (central teeth) to lateral position (laterals I, II, and marginals) due to functional gradients, with the highest wear patterns (tooth loss, tooth volume loss and cusp damages like chipping, fractures, rounding and scratches) in the centre. (b) The mechanical properties of the teeth are related to the degree of interaction with ingesta, with the hardest and stiffest teeth in the centre and with the softest ones on the sides. (c) The mechanical properties are related to the high inorganic content. As a result, (d) specialized coatings as potential wear-coping mechanisms are expected at the sites that are in direct contact with ingesta.

## 2. Material and methods

### 2.1. Preparation of *Loligo vulgaris*

A total of 90 individuals of *Loligo vulgaris* were dissected in 2022 in a bachelor student zoology course at the University of Hamburg and 68 radulae were used for this study. These specimens were ordered from a local fish market and came from the North Sea. For this study, we only used animals of the same size (mantle length of 14–15 cm), because it is known that the buccal mass sizes change with body size [106,121–123].

The whole buccal mass was extracted by forceps and the beaks were carefully removed. The supporting structures were detached from the radular membrane by forceps. To avoid artefacts from sample preparation on the teeth, the radula was only grabbed at the hyaline shields (= alary processus). Each radula was placed in one Eppendorf tube filled with 70% EtOH and cleaned of organic tissues by rinsing with alcohol.

**Figure 2.**
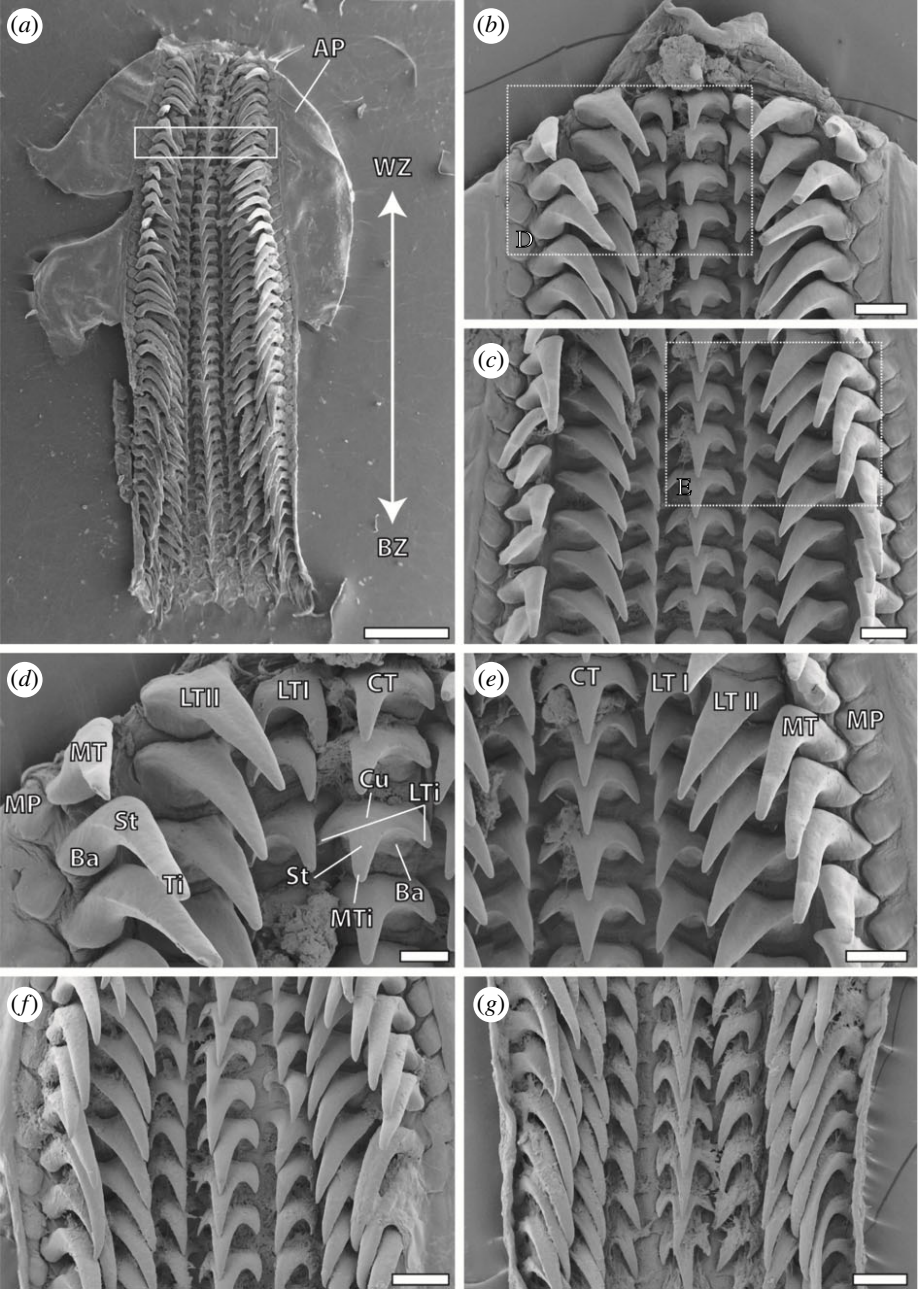
SEM images of *Loligo vulgaris* radula. (*a*) Overview. The white box highlights the region 80–85%; here the teeth were used for EDX, CLSM and nanoindentation. (*b*) Oldest tooth rows in the working zone at higher magnification (*d*). (*c*) Used teeth of the working zone at higher magnification (*e*). (*f*) Mature teeth with no wear from zone 40%. (*g*) Younger teeth from the building zone. Scale bars, (*a*) 1 mm; (*b*,*c*,*e*–*g*) 200 µm; (*d*) 100 µm. AP, alary processus (= hyaline shield); Ba, tooth basis; BZ, building zone; Cu, tooth cusp; LTi, lateral tip of central tooth; LZI, lateral tooth I; LZII, lateral tooth II; MP, marginal plate; MT, marginal tooth; MTi, major tip of central tooth; St, tooth stylus; Ti, tooth tip; WZ, working zone.

**Figure 3.**
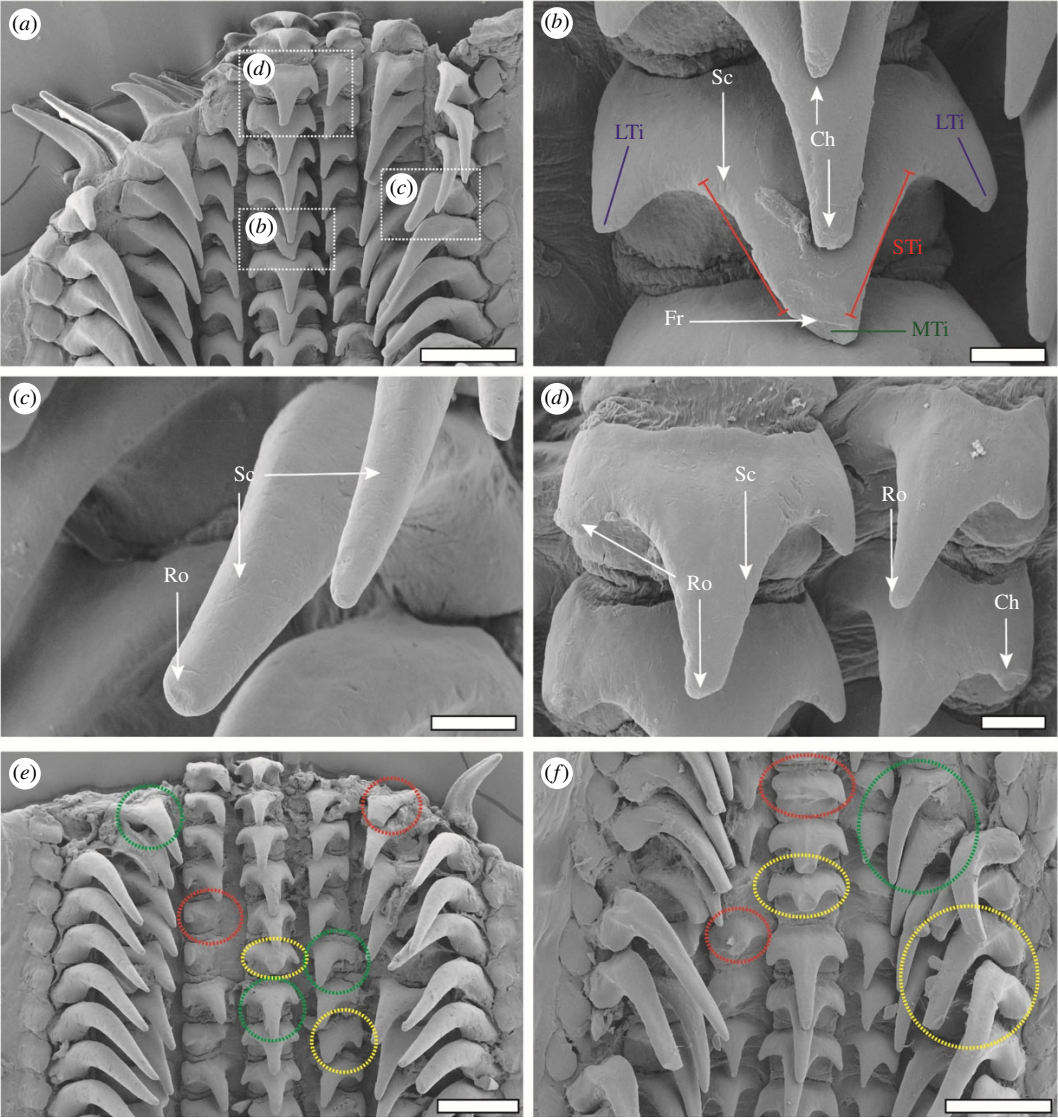
SEM images displaying the characteristic wear types and the tooth volume loss. (*a*) Overview of the working zone. (*b*) Central tooth with scratches, chipping, and fractures. Wear was identified on the major tip, the lateral tips, and the sides of the major tip. (*c*) Marginal tooth with rounding and scratches. (*d*) Central tooth (left) and lateral I teeth (right) with rounding, scratches, and chipping. (*e,f*) Working zones with highlighted tooth volume loss: green = 0–25% loss, yellow = 25–75% loss, red = 75–100% loss. Scale bars, (*a*) 200 μm; (*b*–*d*) 30 µm; (*e,f*) 200 µm. Ch, chipping; Fr, fracture; LTi, lateral tip; MTi, major tip; Ro, rounding; Sc, scratch; STi, side of major tip.

**Figure 4.**
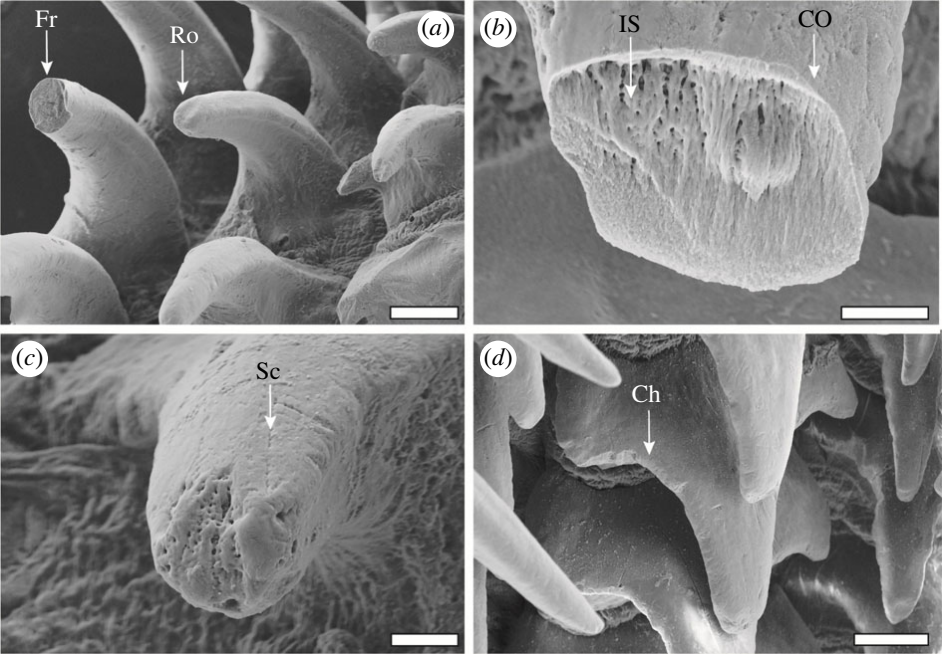
SEM images of the wear at higher magnifications. (*a*) Marginal tooth (left) and lateral tooth I (right) with fracture and rounding. (*b*) Fracture displaying the coating and the inner fibrous structure of the tooth. (*c*) Rounded tip. (*d*) Chipping on the lateral tips and the side of the major tip. Scale bars, (*a*,*d*) 40 µm; (*b*) 8 µm; (*c*) 10 µm. Ch, chipping; CO, coating of the tooth; IS, inner fibrous structure of the tooth; Fr, fracture; Ro, rounding; Sc, scratch.

Radulae were mounted on SEM sample holders with double-sided adhesive carbon tape and sputter-coated with a 5 nm layer of platinum (figure 2). Images were taken using a Zeiss LEO 1525 scanning electron microscope (One Zeiss Drive, Thornwood, USA) with 5 kV. Twenty-two radulae were excluded from the study due to noticeable contamination. The remaining 68 radulae were visualized in the SEM at high magnifications (see figures 3 and 4).

### 2.2. Documentation

Due to similarities in radular and tooth morphology, the teeth were named according to the terminology used for the radula of *Octopus vulgaris* [86]. For each radula, the tooth rows were counted starting from the youngest tooth row in the radular building zone (number 1 was assigned) up to the radular working zone and degeneration zone. Subsequently, for each tooth in a row, the type of wear was documented (see below). For the central teeth, because these were freestanding and wear could be documented in most radular regions, the major tip (MTi in figure 3*b*), the lateral tips (LTi in figure 3*b*), the side of the major tip (STi in figure 3*b*), and the overall surface was examined. For the lateral teeth I, lateral teeth II, and marginal teeth, wear was not determined more specifically to the tooth region, because teeth overlapped and could, thus, not be fully documented using SEM. For the volume loss, we differentiated between the left and right marginal, lateral II, and lateral I. For the wear of the remaining tooth structure (i.e. scratches, rounding, fracture, chipping), we differentiated between ‘*both sides show this type of wear*’ and ‘*only one of the two sides shows this type of wear*’ for the marginals, laterals II and laterals I.

Since the quantities of tooth rows differed between individuals (38–60 tooth rows), we converted the row number to percentages, so that tooth row number 1 (i.e. the youngest one) was equal to 0% and the oldest one to 100%. Then, to enable a better comparison between radular regions, the percentage values were rounded to the next 5%, so that e.g. 4% equalled 5%, 8% equalled 10% and so on. Overall, 12 512 teeth were documented. 11 972 teeth were clean and their wear state could be documented. 535 teeth were full of organic matter and their state could not be documented.

### 2.3. Wear categories

From obtained SEM images, each tooth, which was not covered by organics, was assigned to the following volume categories following the protocol of Shaw *et al*. [4]: 0–25% loss (highlighted by green in figure 3*e*,*f*), 25–75% loss (highlighted by yellow in figure 3*e*,*f*), 75–100% loss (highlighted by red in figure 2*e*,*f*), and being ripped out of the membrane. The wear of each remaining tooth was characterized and sorted to the following four different categories:

(A) *Chipping* was assigned, when smaller parts (less than 5% of the tooth volume) were lost and the remaining edges were sharp (see figures 3*b*,*d*, 4*d*).
(B) *Fractures* were assigned, when a clear loss of a larger tooth fragment (greater than 5% of the tooth volume) with a sharp edge was visible (see figures 3*b*, 4*a*,*b*).
(C) *Rounding* was assigned when material loss could be documented, but the tooth edges were rounded and did not show sharp edges (see figures 3*c*,*d*, 4*a*–*c*).
(D) *Scratches* were assigned when superficial grooves were observable in the SEM images on the tooth's surface (size 1–2 µm) (see figures 3*b*–*d*, 4*d*).

### 2.4. Confocal laser scanning microscopy

For documentation of the structures' heterogeneities, we followed the protocol of Michels & Gorb [124], documenting arthropod cuticle autofluorescence, when exposed to lasers of distinct wavelengths. This procedure is mostly applied to insect cuticle (e.g. [125–129]), but has been previously also applied to chitin structures with higher mineral content such as crustacean cuticle [130–132] and radular teeth [15,22,23,37]. For this purpose, two cleaned radulae were arranged on object glass slides and each one was surrounded by a stack of reinforcement rings. Each stack was filled with glycerine (greater than or equal to 99.5%, free of water, Carl Roth GmbH & Co. KG, Karlsruhe, Germany) and covered with a glass coverslip. Samples (in one radula, the region 60–90% and in another one, the region 80–90% were documented) were then visualized employing a Zeiss LSM 700 confocal laser scanning microscope (Carl Zeiss Microscopy GmbH, Jena, Germany) with four stable solid-state lasers with wavelengths of 405 nm, 488 nm, 555 nm, and 639 nm. Bandpass or longpass emission filters (420–480 nm, greater than or equal to 490 nm, greater than or equal to 560 nm, or greater than or equal to 640 nm) were applied. After scanning, the autofluorescence images were superimposed (applying maximum intensity projection) with the software Zeiss Efficient Navigation (ZEN) (Carl Zeiss MicroImaging GmbH). Finally, the colour blue was assigned to the autofluorescence signal received from the laser with wavelength 405 nm, green to 488 nm, red (50% saturation) to 555 nm and red (50% saturation) to 639 nm.

In unmineralized chitinous structures, (a) sclerotized, stiff material is related to a red signal (to the autofluorescence after excitation with the lasers of 555 and 639 nm), (b) weakly sclerotized chitin to a green signal (laser of 488 nm), and (c) blue signals to high proportions of elastic proteins (laser of 405 nm) [20,21,37,124–129]. In the structures with a higher inorganic content, (a) sclerotized, stiff material is related to a red signal, (b) silica or weakly sclerotized chitin to a green signal, and (c) blue signals to high proportions of elastic proteins or calcium [15,22,23,130–132].

### 2.5. Elemental analysis

To test the elemental composition by EDX/EDS, seven additional radulae were extracted from specimens and cleaned in an ultrasonic bath for 1 min. Then, radulae were attached to glass object slides by double-sided adhesive carbon tape, following our previous protocols [46]. Afterwards, each radula was surrounded by a small metallic ring, which was then filled with epoxy resin (Reckli Epoxy WST, RECKLI GmbH, Herne, Germany) covering the complete radula. After polymerization for three days at room temperature, slides and tapes were removed. Samples were polished with sandpapers of different roughness until the tooth cross-sections were on display. Then, the sample surface was smoothened with aluminium oxide polishing powder suspension of 0.3 μm grainsize (PRESI GmbH, Hagen, Germany) on a polishing machine (Minitech 233/333, PRESI GmbH, Hagen, Germany) to prevent electron scattering. Then, the samples were cleaned in an ultrasonic bath for five minutes to remove the aluminium oxide, mounted on SEM sample holders and sputter-coated with platinum (5 nm layer). The elemental composition was determined with the SEM Zeiss LEO 1525 equipped with an Octane Silicon Drift Detector (SDD) (micro analyses system TEAM, EDAX Inc., New Jersey, USA). For each sample, the same settings were used (i.e. an acceleration voltage of 20 kV, working distance, lens opening, etc.) and the detector was calibrated with copper.

From each individual radula, we tested the mature teeth of three rows from the radular zone 80–85%. For each tooth type, we tested the inner structure of the base, cusp, the stylus of the tip, and the tip. The surface of the teeth (0.2–0.5 µm thickness) was examined at the base, the cusp, the side of the tip and the tip. Additionally, to gain more insight into the origins of the autofluorescence, we tested the inner structure and the surface of the alary processus and the inner structure of the membrane. Overall, 318 small areas (0.2–8.0 × 0.1–3 µm size) were successfully tested by EDX (see electronic supplementary material, table S1, for the numbers of measurements per locality).

The proportions of the following elements were measured: Al (aluminium), C (carbon), Ca (calcium), Cl (chlorine), Cu (copper), Fe (iron), H (hydrogen), K (potassium), Mg (magnesium), N (nitrogen), Na (sodium), O (oxygen), P (phosphorus), Pt (platinum), S (sulfur), Si (silicon) and Zn (zinc). We did not find Fe, K and Zn. Some elements were not discussed as they are either the elemental basis of chitin and proteins (H, C, N, O) or of the polishing powder (Al, O). The single peak of P overlaps with that of Pt. Because of this, the software could not discriminate between these two elements and P content could not be reliably determined. Therefore, P and Pt are discussed together (P + Pt). We, however, measured 15 areas of pure epoxy to obtain some values on the Pt content (mean ± standard deviation: 0.14 ± 0.03 atomic %). Due to this testing, we also know that none of the discussed elements (Ca, Cl, Cu, Mn, Si, Si, Zn, etc.) was present in the epoxy and that this is not an artefact from sample preparation.

### 2.6. Nanoindentation

Nanoindentation experiments, following established protocols [30,40], were performed on the samples from EDX (seven radulae), but only on the inner structure of the teeth, as the surface layer was too thin to perform these experiments. Again, the teeth from the region 80–85% were tested. After the elemental analyses, the teeth of three tooth rows were tested at the base, the cusp, the stylus of the tip, and the tip with a nanoindenter (SA2, MTS Nano Instruments, Oak Ridge, Tennessee, USA) equipped with a Berkovich indenter tip and a dynamic contact module (DCM) head. Overall, 459 localities were successfully tested (see electronic supplementary material, table S1, for the numbers of measurements per locality).

Hardness (*H*) and Young's modulus (*E*) were determined from force–distance curves by applying the continuous stiffness mode. *E* and *H* were determined at penetration depths of 600–800 nm. For each site indented, we obtained approximately 200 values at different indentation depths, which were averaged to afford one *H* and one *E* mean value per indent. All tests were performed under normal room conditions (relative humidity 28–30%, temperature 22–24°C).

Since not all regions of interest were on display at the same section, we tested the surface target localities first by EDX and nanoindentation. After this, samples were smoothened and polished until the next target localities were on display. Then, EDX analyses were performed first and nanoindentation afterwards; this procedure was repeated until all regions of interest were tested.

### 2.7. Statistical analyses

All statistical analyses were performed with JMP Pro, Version 14 (SAS Institute Inc., Cary, NC, 1989–2007). The wear was visualized with bar charts. Mean values and standard deviations were calculated for the results from EDX and nanoindentation analyses. Shapiro–Wilk W tests, to test normality of distribution, were conducted. As the data were non-normally distributed, a Kruskal–Wallis/Wilcoxon test, followed by pairwise comparison with the Wilcoxon method, was carried out. With this, we compared (a) the results of elemental analysis for the inner structure and the coating for each individual structure (tooth position and region or part) and (b) the *E* and *H* within each tooth type. Correlation coefficients between the discussed elements were also calculated.

## 3. Results

### 3.1. Radular morphology

**Figure 5.**
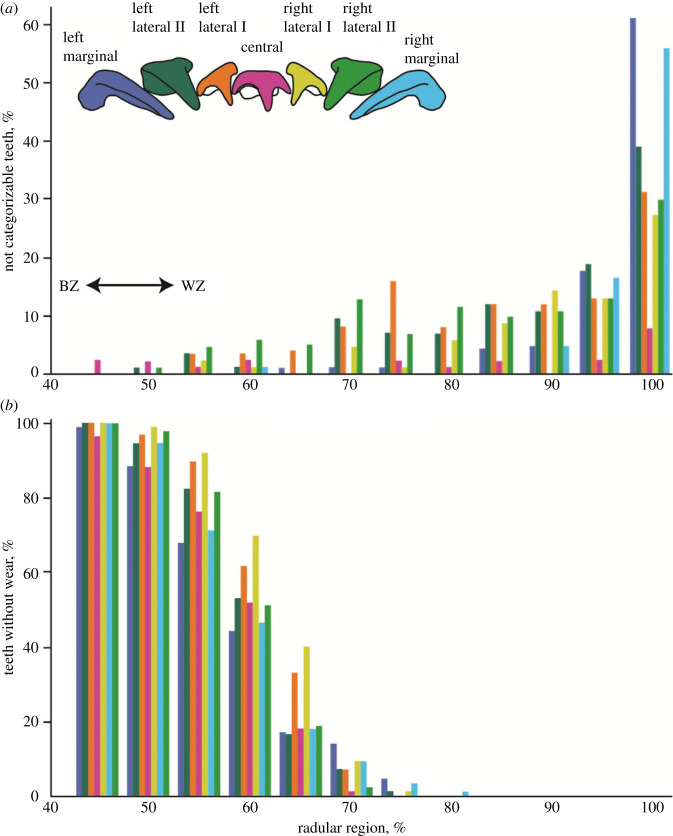
(*a*) Proportions of teeth, given in %, along the radula that could not be categorized, because they were lost (but not ripped out) or were covered with organic matter. In radular region 0–40% all teeth could be categorized. (*b*) Proportions of teeth, given in %, along the radula with no signs of wear. In radular region 0–40% all teeth showed no wear. The colours of the bars relate the tooth type. BZ, building zone; WZ, working zone.

The quantity of tooth rows varied between 38 and 60 (45 rows per specimen were found on average). In the building zone (figure 2*g*), teeth were immature and unused. First signs of wear appeared at 45% of the radular length, which in turn showed that less than 55% of the entire radula were used and part of the working zone (see figure 5 and electronic supplementary material, tables S2–S5, for the wear sorted to each tooth type and radular region).

There were four different tooth types per row on the transparent membrane (figure 2). The central tooth was medial, flanked to each side by one lateral I, one lateral II, one marginal tooth, and one marginal plate. The central tooth bore one pronounced major tip, which was flanked to each side by one smaller lateral tip (see figures 2*d*, 3*b*,*d*). The lateral tooth I was morphologically similar to the central tooth, but due to curvature of the membrane, the wear on the lateral tips of the lateral teeth I could not be identified in most rows (figure 3*d*). Therefore, only the wear on the major tooth tip of the lateral I was documented. The lateral II tooth and the marginal tooth possessed long and prominent tips, but lacked lateral tips (see figures 2*e* and 3*c*).

### 3.2. Uncategorizable teeth

Overall, we could see and document 11 972 teeth. 535 teeth were not categorizable (80 left marginals, 98 left laterals II, 97 left laterals I, 22 centrals, 72 right laterals I, 95 right laterals II and 71 right marginals). Most teeth that could not be sorted to a category due to their loss were primarily found in the outermost, oldest region (region 100%) (see figure 5 and electronic supplementary material, tables S6–S8, for the number of uncategorizable teeth, sorted to each tooth type and radular region). Particularly, the marginal teeth had a very high percentage of uncategorizable teeth (55–60% in region 100%), followed by the lateral II, lateral I, and finally the centrals with the lowest number of teeth that could not be categorized (figure 5 and electronic supplementary material, tables S6–S8).

### 3.3. Teeth without wear

Up to radular region 45%, 100% of the teeth did not show wear (see figure 5 and electronic supplementary material, tables S2–S5, for the wear sorted to each tooth type and radular region). The central teeth and the marginals experienced wear first, followed by the laterals II, and finally the laterals I. It is striking how rapidly the amount of unused teeth decreased from region 55% onwards. After region 65%, more than half of all evaluated teeth from all tooth types showed wear. The oldest unused teeth, some right marginals, were found in region 80% (figure 5). The left and the right teeth show different degrees of wear, which indicates that the radula interacted with the food asymmetrically.

### 3.4. Tooth volume loss and wear

#### 3.4.1. Tooth volume loss

**Figure 6.**
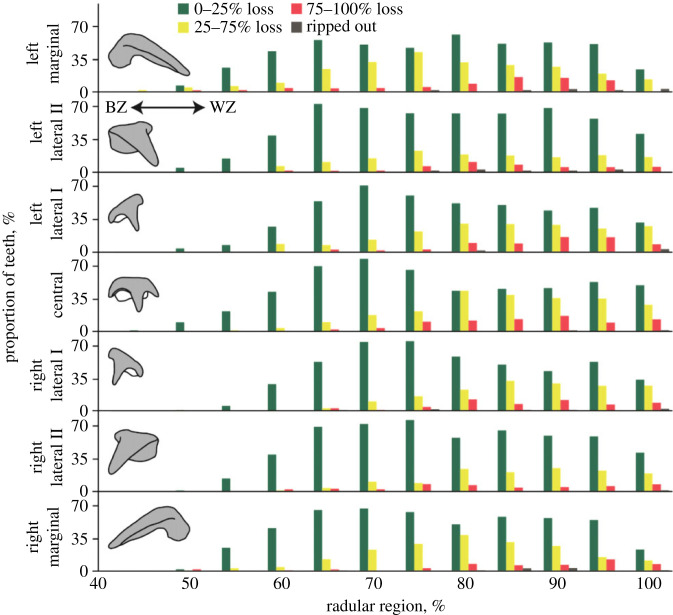
Wear. Tooth volume loss, given in %, for each tooth type along the radula. In region 0–40% no volume loss was documented.

The volumes of all teeth were reduced during ontogeny (see figure 6 and electronic supplementary material, tables S6–S8, for the volume loss sorted to each tooth type and radular region). The volume loss was first documented in the region 45% for the central tooth, whereas the volumes of all other teeth were reduced from the region 50% on. Low volume loss (up to 25% loss; figure 3*e*,*f*) was most common on the radula, whereas a complete loss of the tooth (ripped out of membrane) was the rarest case (detected from the region 65% on). With the higher radular region, the proportions of teeth with moderate and high tooth volume loss increased (figure 6 and electronic supplementary material, tables S6–S8). After the region 75–80%, the values for tooth volume loss decreased, which can be explained by the increasing number of teeth that could not be categorized. For all tooth types, the volume loss was rather similar and there was no clear pattern of the volume loss.

#### 3.4.2. Wear

**Figure 7.**
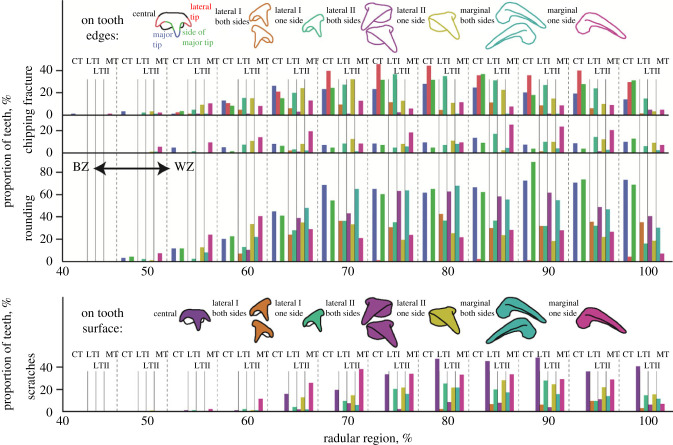
Wear documented for all tooth types and regions along the radula. Rounding, fracture, and chipping are related to the edges of the teeth, whereas scratches refer to the tooth surface. For the marginal, laterals II and laterals I, we differentiated between the categories ‘*both sides show this type of wear*’ and ‘*one side shows this wear*’. For the edges of the central tooth, we differentiated between the major tip, the lateral tips, and the side of the major tip. The colours of the bars correspond to the tooth type and the distinct categories or tooth edges. In region 0–40%, no wear was documented. BZ, building zone; CT, central tooth; LTI, lateral tooth I; LTII, lateral tooth II; MT, marginal tooth; WZ, working zone.

As observed under SEM, the amount of teeth with all four kinds of wear increased during ontogeny (see figure 7 and electronic supplementary material, tables S2–S5, for the wear sorted to each tooth type and radular region). From region 95% on, the amount of teeth with wear decreased, which can be explained with the higher number of lost and thus uncategorized teeth, especially in the region 100%.

#### 3.4.3. Wear on the central teeth

Chipping could be detected earliest in ontogeny (from region 45% on; figure 7 and electronic supplementary material, table S2). It was mostly determined on the major tips. From region 55% on, this kind of wear appeared on the sides of the major tip and on the lateral tips. In general, we found that the sides of the major tips experienced most chippings, followed by the lateral tips and finally the major tips. During ontogeny, rounding appeared at the same region (from region 50% on; figure 7 and electronic supplementary material, table S2) on the major tips and the lateral tips. Rounding on the sides of the major tips, which also reduced the width of the major tips, appeared least and was documented only in very worn radular teeth (from region 85% on). Fractures were documented first on the major tips and least on the lateral tips. Scratches were found from region 55% on (figure 7 and electronic supplementary material, table S2).

#### 3.4.4. Wear on the marginals, laterals II and laterals I

In these teeth, wear appeared later (from region 50% on; figure 7 and electronic supplementary material, tables S3–S5) than in the central teeth. Fractures, chippings, scratches and rounding could all be documented from this region on. Interestingly, the marginal teeth were instantly more affected from wear than the laterals. With regard to fractures, scratches, and rounding across most of the radular length, a functional gradient was found; the marginals were affected most, followed by the laterals II, and finally the lateral I. With regard to chipping, the laterals I seem to be affected most, followed by the laterals I, and finally the marginals. Wear on only one side of the radula appeared first during ontogeny (figure 7 and electronic supplementary material, tables S3–S5).

### 3.5. Autofluorescence

**Figure 8.**
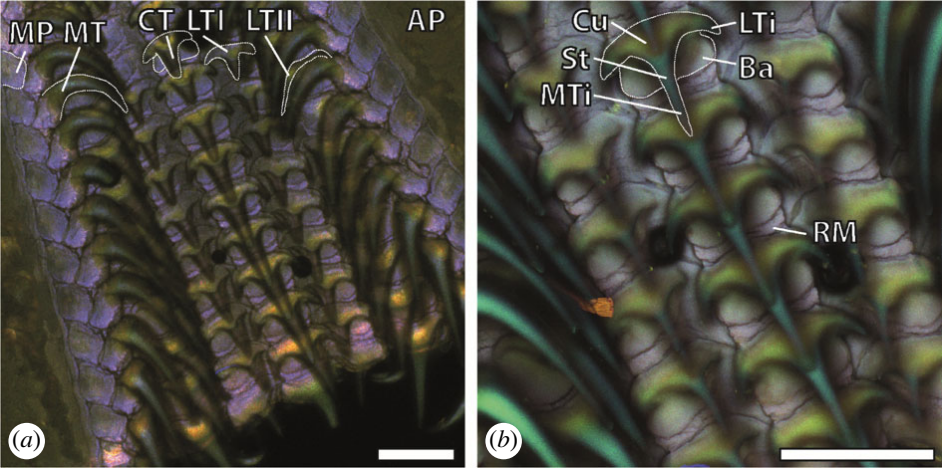
CLSM images of one radula showing the heterogeneities of the material. (*a*) Overview of region 60–90%. (*b*) Magnification of region 80–90%. Scale bars, 180 μm. AP, alary processus (= hyaline shields); Ba, basis; CT, central tooth; Cu, cusp; LTI, lateral tooth I; LTII, lateral tooth II; LTi, lateral tip; MP, marginal plate; MT, marginal tooth; MTi, major tip; RM, radular membrane; St, stylus.

The tooth bases and parts of the marginal plates emitted strong autofluorescence signals after excitation with the lasers of 405, 555 and 639 nm wavelengths (the blue colour was assigned to the autofluorescence from 405 nm and the red colour to that from 555 and 639 nm) (figure 8). The membrane emitted a strong autofluorescence signal after excitation with the laser of 405 nm wavelength (the blue colour was assigned to this wavelength) and some signal from 555 and 639 nm (red colour), which appear violet in overlay. The tooth tips emitted a strong autofluorescence signal after excitation with the 488 nm laser (the green colour was assigned to this autofluorescence) (figure 8).

### 3.6. Elemental composition

#### 3.6.1. Inner fibrous structure of teeth

**Figure 9.**
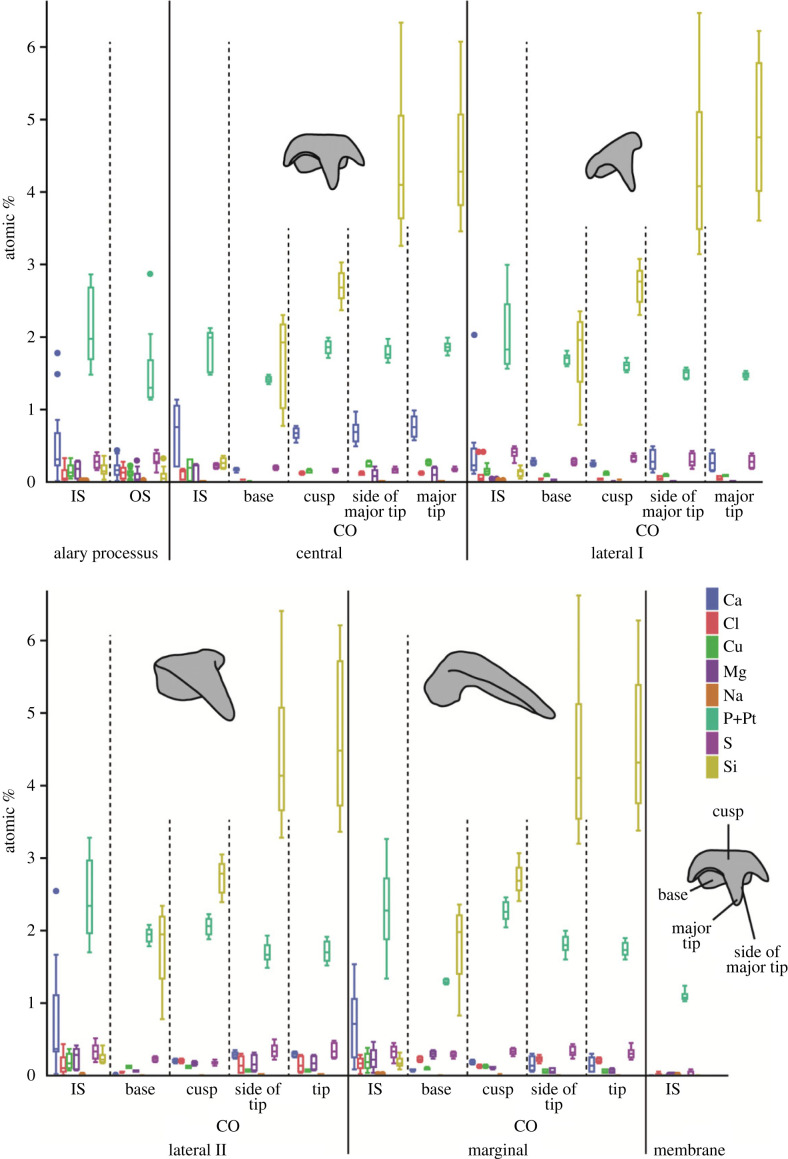
Results from EDX analyses for each tooth type, the alary processus, and the radular membrane in the region 80–85%. The results for the inner structure, the surface or coating are depicted. CO, coating of teeth; IS, inner region of the structure; OS, outer surface of the structure.

In the inner structures of the teeth, P + Pt was determined at highest proportions (means between 1.10 and 2.43 atomic percent), followed by Ca (0.42–0.70), S (0.23–0.40), Si (0.14–0.27), Mg (0.03–0.25), Cu (0.16–0.19), Cl (0.12–0.17) and Na with the lowest proportions (0.01) (figure 9 and electronic supplementary material, table S1). All these values are low. In the alary processus, these elements were determined in small proportions as well. Here, P + Pt was detected most (mean ± standard deviation: 2.12 ± 0.50 atomic percent), followed by Ca (0.51 ± 0.49), S (0.28 ± 0.08), Si (0.18 ± 0.08), Mg (0.16 ± 0.11), Cu (0.16 ± 0.08), Cl (0.10 ± 0.09), and finally Na with the lowest proportions (0.01 ± 0.01). In the membrane, Ca, Cu, Mg, Si were not detected; here, contents of Cl (0.01 ± 0.02), Na (0.01 ± 0.01), S (0.02 ± 0.03) were very low, in contrast to P + Pt (1.1 ± 0.06) (figure 9 and electronic supplementary material, table S1).

#### 3.6.2. Surfaces of the structures

With regard to the surface of the structures (i.e. coatings), Si was determined with highest proportions (means between 3.31 and 3.44 atomic percent), followed by P + Pt (1.57–1.84), Ca (0.14–0.58), S (0.18–0.31), Cl (0.05–0.20), Cu (0.09–0.18), Mg (0.02–0.14), and Na with the lowest proportions (0.01) (figure 9 and electronic supplementary material, table S1). All these values, except for Si and P + Pt, are low.

In the alary processus, these elements were determined as well. Here, P + Pt was detected most (mean ± standard deviation: 1.51 ± 0.47 atomic percent), followed by S (0.30 ± 0.09), Ca (0.19 ± 0.11), Cl (0.13 ± 0.08), Cu (0.13 ± 0.05), Si (0.08 ± 0.09), Mg (0.07 ± 0.09), and finally Na with the lowest proportions (0.01 ± 0.01) (figure 9 and electronic supplementary material, table S1).

Si content was always significantly higher at the surface than in the inner structure (see electronic supplementary material, table S9, for *p*-values). Ca, Cu, Mg, and P + Pt contents were mostly significantly higher in the inner structure than at the surface. For the rest of the discussed elements, no clear pattern could be found.

#### 3.6.3. Correlation coefficients of elements

The elements Cu and Ca correlated highly positive (*r* = 0.70). P + Pt and Cu (0.59), Cl and Mg (0.53) showed moderate positive correlations. Ca and P + Pt (0.47), P + Pt and Mg (0.40), Cu and Mg (0.34) correlated lowly positively. Ca and Mg (0.29), Ca and Cl (0.28), Cl and P + Pt (0.26), S and P + Pt (0.22), Cu and Na (0.20), Cu and Cl (0.19), P + Pt and Na (0.19), Ca and Na (0.16), Cl and Si (0.16), S and Na (0.13), Cl and S (0.12), S and Mg (0.09), S and Si (0.05), Na and Si (0.03), Ca and S (0.03) and Cu and S (0.00) showed negligible correlations. Mg and Na (−0.01), Ca and Si (−0.02), Cu and Si (−0.04), Si and Mg (−0.09), P + Pt and Si (−0.14), and Cl and Na (−0.14) showed negative negligible correlations.

### 3.7. Mechanical properties

**Figure 10.**
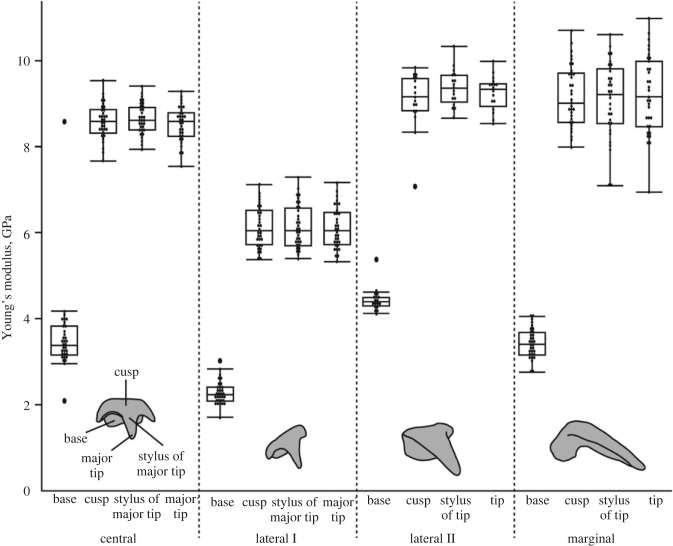
Young's modulus (*E*) values (in GPa) from nanoindentation testing for each tooth and its parts from region 80–85%.

Both parameters *E* and *H* showed a very high positive correlation (*r* = 0.97). The mean values of *E* ranged from 2.29 to 9.36 GPa and the mean values of *H* between 0.07 and 0.38 GPa (figure 10 and electronic supplementary material, table S1). The marginals were the hardest and stiffest teeth, followed by the laterals II, the centrals, and finally the laterals I as the softest and most flexible teeth. In all teeth, the bases were significantly softer and more flexible than the cusp, the stylus, or the tip (*p* < 0.0001; for *p*-values see electronic supplementary material, table S10). The latter three parts were not significantly different within each tooth.

## 4. Discussion

### 4.1. Limitations

Our results for the first time provide detailed descriptions of the wear types on *Loligo vulgaris* radular teeth, which can be used to compare and characterize different types of radulae in the future. However, the wear was documented two-dimensionally employing SEM, which leads to some data loss, as wear is always three-dimensional. It would be advantageous for future studies to employ detailed dental surface texture analysis, a method that quantifies entire surfaces in three dimensions and represents them as a three-dimensional point cloud (see [1,3,13,133–136]). This is however rather difficult as radular teeth are small and roundish.

Another problem during documentation of the tooth wear was the arrangement of the teeth on the membrane, since teeth overlap one another. In most radulae, the central teeth could be documented entirely in the SEM, which was, however, in contrast to the laterals I, II and marginals, whose tips could be well documented. Despite the limitations in methodology, the obtained data and the associated conclusions can serve as a basis for further research.

### 4.2. The origin of wear

Previously, there have been few attempts to correlate the parameters of radular teeth (morphology, mechanical properties, chemical composition, tooth arrangement on the membrane) to the observed wear and the ingesta [4–6,137]. In Shaw *et al*. [4], the tooth volume loss was documented systematically for the highly mineralized teeth of Polyplacophora and Patellogastropoda, documenting the decrease in tooth volume during radular ontogeny. Krings & Gorb [6] determined that the amount of heterobranch gastropod teeth used in feeding positively correlates to the abrasiveness of the ingesta, thus using wear to identify the functional radular area. In Krings *et al*. [5] on the neritid gastropod *Vittina turrita*, the wear from controlled feeding experiments was used to reveal the three-dimensional arrangement of the teeth on the radular membrane during feeding. Ukmar-Godec *et al*. [137] described the wear on limpet teeth to detect self-sharpening mechanisms. However, there is a profound lack of knowledge with regard to the wear on radular teeth.

In general, there is, as expected (hypothesis a), a progressive wear towards the anterior end of the radula in *Loligo vulgaris*, starting from region 45% on, which shows that most parts of the radula either interact with the ingesta or are already degenerating.

*Loligo vulgaris* has a broad spectrum of prey, including crustaceans, fish, polychaets and other cephalopods [53–60]. This predatory feeding strategy is reflected in the morphology of the teeth. The teeth of *L. vulgaris* possess small and thin tips, which are likely used for piercing or cutting due to a smaller contact area that can transfer higher pressures during foraging (for review on puncture mechanics, see [138]). In general, the intimate interaction between teeth and ingesta has a significant impact on the wear on the teeth. Even though wear is influenced by several parameters, such as (1) the speed of contact between tooth and foreign body, (2) the load, (3) the stiffness/hardness of the materials, (4) the wetting degree, and (5) the temperature [139], it results from the mechanical interactions between tooth, ingesta, and sometimes third bodies, such as lubricants and abrasive particles [140–142]. Based on the latter, it is possible to propose some basic hypotheses about the kind of interaction by analysing wear patterns of one surface.

**Figure 11.**
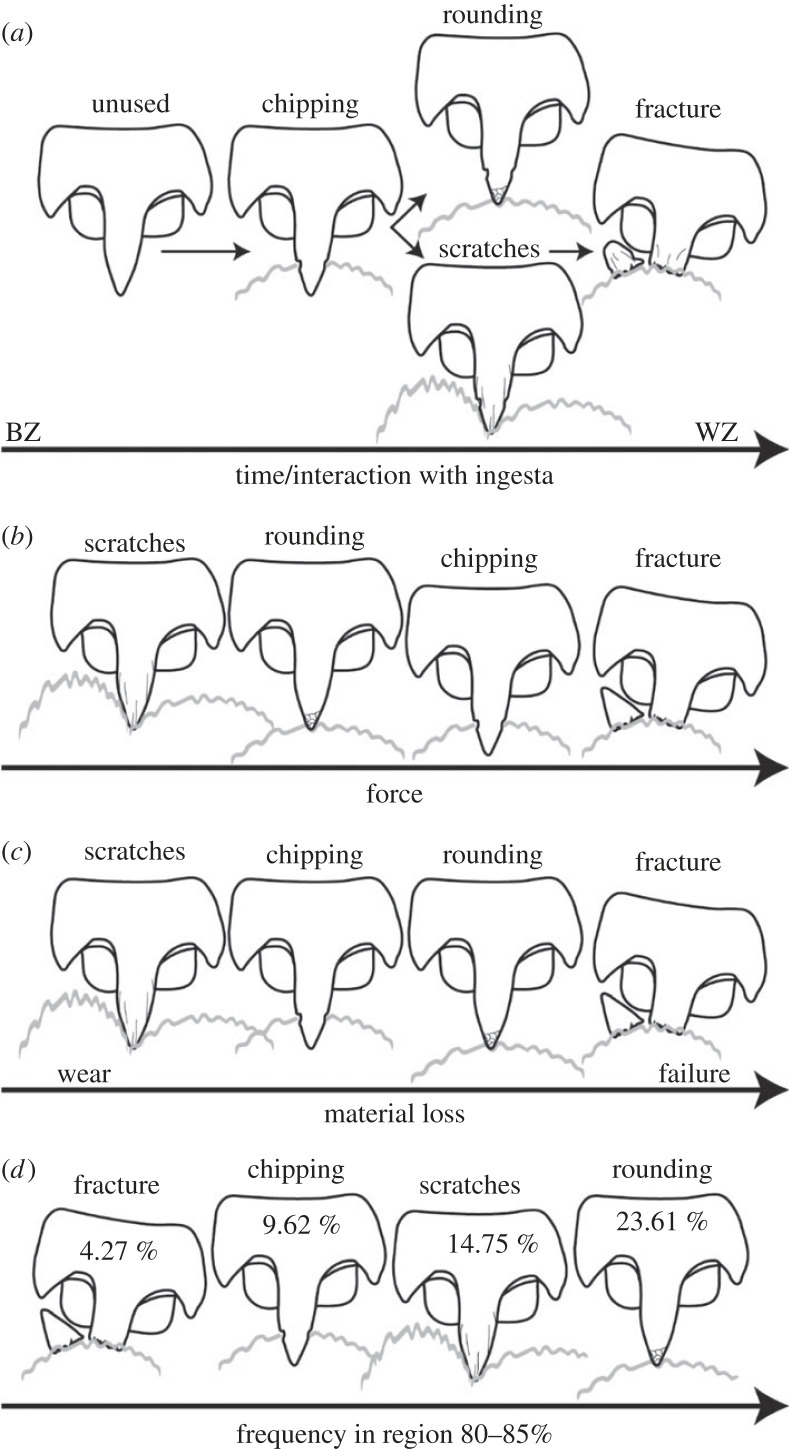
(*a*) Appearance of wear during ontogeny. (*b*) Hypotheses on the origins of the wear with regard to the force involved. Fracture: teeth are brought with highest force onto a hard and stiff ingesta surface (e.g. the carapace of a crustacean). This results in structural failure on the thin stylus of the tip. Chipping: teeth puncture a hard and stiff ingesta (e.g. the carapace of a crustacean), which exhibit sharp edges when breaking, leading to chipping on the stylus of the tip. Rounding: the tip is repeatedly moved across an abrasive surface (e.g. the carapace of a crustacean or fish scales), which rounds the tooth surface. Scratches: with least force, teeth are moved across an abrasive surface (e.g. the carapace of a crustacean or fish scales), leaving scratches on the tooth surface. (*c*) Relative material loss for the different types of wear. (*d*) Frequency of the wear types from the region 80–85%. The numbers in the teeth refer to the proportions of the teeth from this region that are affected by this type of wear.

Scratches are the first indicator and result of lower material loss, followed by chipping, rounding and finally fractures with the most material loss (figure 11*c*). One could hypothesize that the minimal material loss (scratches) would appear first and the maximal (fracture) last during ontogeny, but surprisingly this was not the case (figure 11*a*). Chipping was documented in the younger teeth, whereas rounding and scratches appeared in older teeth, but at the same time. Fractures occurred, as expected, latest during ontogeny. This could potentially be explained by the behaviour of the squid, i.e. the force involved in the interaction with the ingesta (figure 11*b*). Scratches on the tooth surface are likely the result of an interaction between coarse and sharp parts of the prey (carapaces of crustacean, fish bones, etc.) with relatively small forces involved. A more forceful interaction probably leads to small fractures of the tooth tip, which finally, after multiple interactions lead to the rounding of the tip. Chipping could be the result of interaction with sharp and hard edges, which could originate from the punctuation of thicker crustacean carapaces requiring comparably more force. If the force during interaction is too high, fractures can occur (figure 11*b*). The latter wear can surely also be the result of an interaction at high speed or of an interaction with hard ingesta surfaces. Additionally, teeth that are already heavily worn are likely to fracture, as cracks expand more during each stress cycle leading to both fracture and material loss. Since, as mentioned above, chipping appears first during ontogeny, it is likely that the main tooth–ingesta interaction is the puncturing of exoskeletons. Teeth that already display some chipping could, through further interactions, break. In turn, this fracture could, with more puncturing events, become rounded (and can thus not be recognized as fracture). This latter hypothesis is supported by the observation that in the region 80–85% rounding is the most common type of the wear, followed by scratches, chipping and finally fracture (figure 11*d*). All these thoughts are first approximate explanations that require more detailed research, potentially involving feeding experiments. Additionally, the force needed to cause the specific types of the wear should be documented, potentially in the form of breaking-stress experiments (e.g. similar to [15,34,35]).

The wear of the radular teeth is likely also related to the type of prey. The prey of cephalopods is various [53–60] and abrasive ingesta may include fish scales, bones or crustacean carapaces. For future research, it would be interesting to relate the wear patterns of the radula with the stomach contents of the same individual, maybe in the form of controlled feeding experiments. This could provide more insights into the relationship between wear and food. However, the wear of the radula is probably related to the food of the last days or weeks, as the whole radula is probably not replaced daily. This means that the food of at least the last weeks should be known for such study. It would be additionally important to compare the tooth wear across different age groups *of L. vulgaris*, as dietary preferences can change in the course of their lives [143,144].

**Figure 12.**
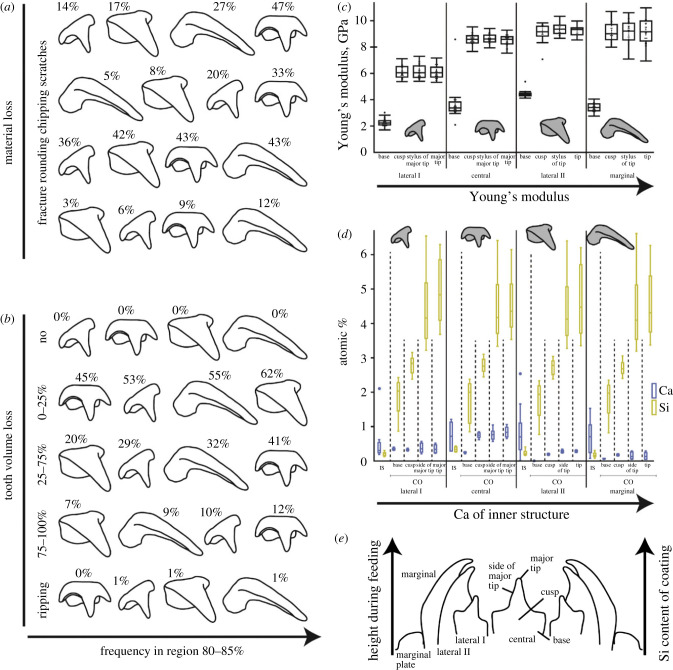
Summary of results. (*a*) Frequency of the different types of wear sorted to each tooth type from the region 80–85%. The numbers (rounded to whole numbers) refer to the proportions of the specific teeth from this region that are affected by this type of wear. (*b*) Frequency of the volume loss sorted to each tooth type from the region 80–85%. The numbers (rounded to whole numbers) refer to the proportions of the specific teeth from this region that are affected by this type of volume loss. (*c*) *E* values of different tooth types, sorted from the most flexibles tooth (lateral I) to the stiffest tooth (marginal). (*d*) Si and Ca content of different tooth types. Teeth are sorted from flexible (lateral I) to stiff (marginal). One can see that the Ca content of the inner tooth structure seems to increase, which could be related to the higher *E* values. With regard to the coating, it can be seen that the Si content increases from the basis to the tip. (*e*) Height of the teeth during feeding as inferred from SEM images (figure 13). The marginal is the highest tooth, followed by the lateral II, the central and finally lateral I. The height of the tooth region during feeding seems to correlate to the Si content of the coating. Abbreviations: CO, coating of teeth; IS, inner region of the structure.

**Figure 13.**
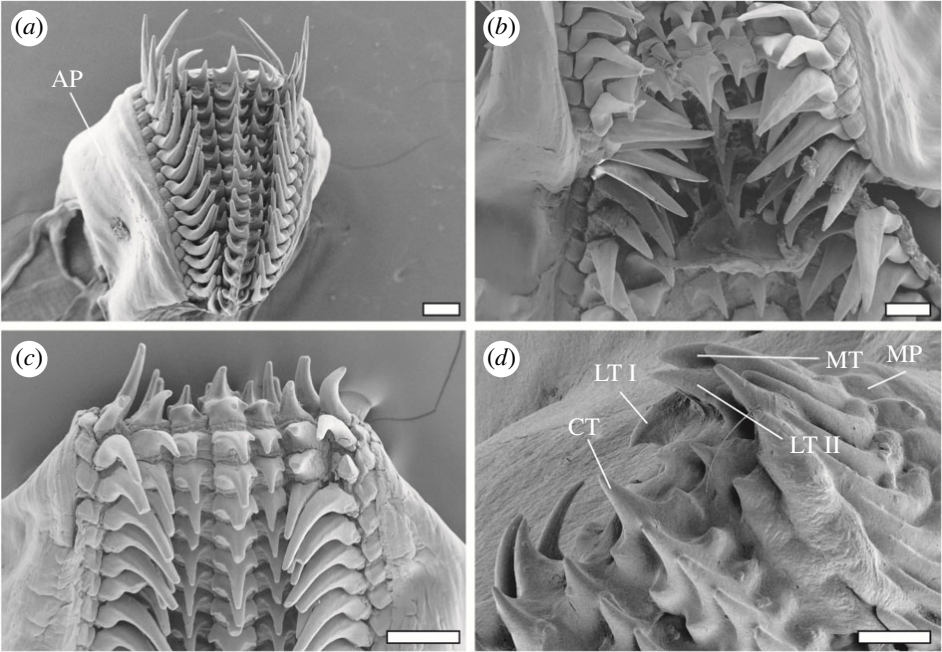
(*a–d*) Natural orientation of the teeth, when the alary processus is not ripped and the teeth are bent along the membrane by the alary processus (proposed feeding position). In (*b*,*d*), one can see that the marginal tooth can interact with the lateral II, potentially distributing stresses. The marginal plates seem to support the marginal teeth to the sides. Scale bars, 200 µm. AP, alary processus (= hyaline shield); CT, central tooth; LTI, lateral tooth I; LTII, lateral tooth II; MP, marginal plate; MT, marginal tooth.

We here detected that the tooth types are affected differently by wear. This verifies our hypothesis (a) ‘the wear patterns differ between the tooth positions'. Additionally, we found that the surfaces that seem to have intimate contact with the ingesta have a higher degree of the wear, which verifies the hypothesis ‘wear can be found in the surfaces that have intimate interaction with the ingesta’. During radular ontogeny, wear appears first at the marginals and central teeth, followed by the laterals II and finally laterals I. This suggests that the marginals and centrals interact with the ingesta first. Additionally, the marginal teeth experience the highest rate of fractures and rounding (figure 12*a*). The highest amount of volume loss, scratches, and chipping occurs on the central teeth (figure 12*a*,*b*). This observation is in congruence with the arrangement of the teeth on the membrane (figure 13): the radula is bent along its longitudinal axis due to the alary processus and the radular supporting structures (see also [64,74,75,81,85–94]). This leads to the situation that the long marginal teeth are situated above the other teeth, followed by the centrals, laterals II and finally laterals I as the shortest and closest teeth to the membrane (figure 13). The marginal teeth likely interact with the food first, followed by the centrals. The central teeth first exhibit wear at the major tips, whereas the wear at the lateral tips occurs later. The major tips are situated above the lateral tips and therefore initially interact with the food. When the major tips fracture or are worn down, the lateral tips are probably more frequently used and experience more wear. Since the marginals probably experience the highest stresses and are thus prone to wear and structural failure, the higher *E* and *H* values of the marginal tips could be an adaptation to reduce the material loss. The laterals I probably interact last and least with the ingesta, which could explain the lower *E* and *H* values of their tips and the low quantity of these teeth that show scratches on their surface. In a previous study on an octopus, Runham *et al*. [89] determined first signs of wear on the central teeth, which were related to the elevated position of the central teeth in comparison with the other ones. This indicates that radulae of different species are bent during the feeding process in a different way and thus show different wear patterns.

### 4.3. Mechanical properties and their origins

We found that the tooth types have different *E* and *H* values that are related to the degree of interaction with the ingesta. This verifies our hypothesis (b) ‘the mechanical properties of the teeth relate to the degree of interaction with the ingesta’. Additionally, we detected that the teeth do not possess high inorganic content, which rejects our hypothesis (c) ‘the mechanical properties are related to high inorganic content’.

The mechanical properties of materials directly contribute to the mechanical behaviour of biological structures. The Young's modulus (*E*) is the measure of the tensile or compressive stiffness and relates to the ability of a material to transmit force [145–148], the mechanical behaviour of the material while puncturing and its resistance to failure (e.g. [149]; for review on puncture mechanics, see [138]). The hardness (*H*) is the measure of the resistance to local plastic deformation induced by indentation or abrasion. Since molluscan teeth are exposed to different forces and ingesta types while foraging, the teeth experience distinct loads [5,6,150–153], which are reflected by their mechanical properties.

Only few molluscan taxa were studied with regard to the mechanical properties of their radular teeth and we are confronted with large gaps of knowledge. However, from the data available, it seems that the *E* and *H* values, the presence or absence of gradients within each tooth and each tooth row, together with the mechanical behaviour of the teeth in concert are related to the tooth's specific function and the species' ecological niche. The mechanical properties, as tested in a species flock of paludomid gastropods, are found to be rather adaptive, whereas the tooth morphology seems more phylogenetically fixed [39,40,152,153]. In general, the mechanical properties can have their origin in the amount of inorganic contents (e.g. iron oxides and silica in Polyplacophora and Patellogastropoda; e.g. [4,8–18]), in the degree of tanning (e.g. some paludomid, nudibranch or heterobranch gastropods; see [22,23,37]), or in the amount of cross-linkers between the chitin fibres (e.g. Ca and Mg in the vetigastropod *Megathura crenulata*; see [29]).

As previously determined for paludomid gastropods, species feeding on soft ingesta (i.e. algae from soft feeding substrate such as sand or mud) possess softer and more flexible teeth (*E* ≤ 8 GPa, *H* ≤ 1 GPa) without clear and pronounced gradients from the tooth basis across the stylus to the cusp [40]. Such soft teeth are probably not capable of transferring higher forces onto the ingesta without structural failure, but possess an increased ability to deform, bend and twist, reducing the risk of failure and allowing the gathering of particles from the substrate. Since the mature teeth within one row have similar mechanical properties, all of them probably serve similar functions (‘monofunctional radula’; see [39,40]). Species feeding on solid ingesta (representatives of Polyplacophora, Fissurellidae, Patellogastropoda and paludomid gastropods foraging on algae covering rocks) or have some interactions with hard ingesta (the nudibranch gastropods (*Felimare picta* and *Doris pseudoargus*) feeding on Porifera with hard spiculae; the heterobranch gastropod *Gastropteron rubrum* feeding on Foraminifera) have significantly harder and stiffer teeth. The dominant teeth of the Polyplacophora are the hardest and stiffest tested radular structures with *E* values ranging from 30 to 130 GPa and *H* from 4 to 12 GPa; here the high content or inorganic material between the chitin fibres is presumably responsible for the elevated *E* and *H* values [11,15,27]. The dominant teeth of *Patella vulgata* (Patellogastropoda) are also very hard and stiff due to the incorporation of silica (*E*: 52–150 GPa; *H*: 3–7 GPa; see [17,18]).

Less mineralized teeth are, in contrast, more flexible, as e.g. the teeth of the vetigastropod *Megathura crenulata* (*E*: 16 GPa; see [29]). Here, chitin fibres are cross-linked by Ca ions and Mg ions [29]. Nudibranchia teeth, which also contain low inorganic content, show *E* values ranging from approximately 5 to 15 GPa and *H* values from approximately 0.1 to 0.9 GPa [23]. The less mineralized teeth of the heterobranch gastropod *G. rubrum* and the paludomid gastropods foraging on solid ingesta are rather soft and flexible in comparison with the mechanical properties measured in other species (*G. rubrum: H* ∼ 0.1–1.0 GPa and *E* ∼ 1–17 GPa; paludomids: *H* ∼ 0.4 GPa and *E* ∼ 8 GPa; see [22,37,40). These latter teeth are, however, capable of interacting between the rows, resulting in a redistribution of stress from one tooth to another, which enables them to resist the same forces as the highly mineralized teeth of Polyplacophora (‘collective effect’; see [15,33,34]). The teeth of all solid ingesta feeders show pronounced gradients in *E* and *H* along each tooth, with the cusp (especially the leading edge) as the hardest and stiffest part, followed by the stylus, and finally the basis as the softest and most flexible part. The origins of these mechanical property gradients are either the amount of the inorganic content (Polyplacophora, Patellogastropoda), the water content (Paludomidae), the degree of tanning (the nudibranch gastropods *F. picta* and *D. pseudoargus*, *G. rubrum*, some Paludomidae), or the content of cross-linkers (the vetigastropod *Megathura crenulata*) [11,15,17–19,22,23,27,29,34,35,37]. The high *E* and *H* values allow the cusps or tips to puncture the ingesta or to scratch across solid surfaces with the possible formation of local stress at the cusps, but without high degrees of wear or structural failure. The softer and more flexible stylus, together with the basis, provides flexibility and acts as a shock absorber against mechanical impacts [15,22,23,28,30,34,35,37,38,40]. In some of these molluscs (the nudibranch gastropods *F. picta* and *D. pseudoargus*), the teeth of each row have similar mechanical properties. Here, teeth again seem to have similar functionalities (‘monofunctional radula’ [23]). However, in other taxa, there can be pronounced gradients within each transversal tooth row, i.e. the different tooth types can have different mechanical properties. In some paludomid gastropods foraging on solid ingesta, the central teeth are the stiffest and hardest elements, followed by the lateral, and finally the marginal teeth [30,37,40,152,153]. The central and lateral teeth are probably capable of loosening algae from rocks, whereas the marginal teeth rather collect loosened food particles in a complex motion afterwards (labour division within one tooth row). In Polyplacophora and limpets, the dominant tooth is especially hard and stiff in contrast to the central, lateral and marginal teeth [15]. Here, this tooth type has a tight interaction with the rock, whereas the other teeth probably gather the loosened food particles and drag them into the mouth cavity. In *G. rubrum*, teeth seem to have different functions as well, i.e. some holding the ingesta, whereas others serve as bolster [22]. Since teeth of one row can have different functions, this type of radula was previously termed ‘multifunctional radula’ (see [39,40]).

The *E* and *H* values of the teeth from *Loligo vulgaris* are comparable to those from nudibranch gastropods piercing Porifera tissues. The teeth of *L. vulgaris* consist of a softer and more flexible basis and a stiff and hard tip (figure 10). This mechanical property gradient along each tooth was previously also proposed on the basis of the preservation of fossil cephalopod teeth [92]. The bases probably serve as shock absorber against mechanical impacts, comparably to teeth of species foraging on hard ingesta. The mechanical properties of the tips probably increase the formation of local stress while puncturing hard ingesta such as crustacean carapaces or cutting flesh. The marginal teeth are the hardest and stiffest teeth, followed by the lateral teeth II, the centrals, and finally the lateral teeth I as the softest and most flexible teeth. These gradients within the tooth row indicate that the teeth experience different loads during foraging, with the marginals probably experiencing highest forces, followed by the laterals II, the centrals, and finally the laterals I. This observation is in congruence with the above-mentioned wear patterns, as the marginals show the highest degree of fracture and rounding (figure 12*a*) and likely interact more with the ingesta. From SEM images of the three-dimensional position of the teeth during foraging, the laterals I seem to have less interaction with the ingesta, since their tips are in the closest position to the membrane. These teeth are the softest and most flexible ones in the row, which supports this hypothesis. From SEM images (figure 13), we also got the impression that the marginals and laterals II can rely on one another during some cutting action (see also [74]). Potentially, in some cases, these two teeth function as one functional unit. This explains the higher *E* and *H* values of lateral II (because it has interaction with the ingesta) and the low degree of wear and volume loss (figure 12*a*,*b*) (because it is supported by the marginals). From the laterals II, across the marginals to the marginal plates and the membrane, the stress could be redistributed (see also [154,155]). This collective effect, however, awaits further investigation using breaking-stress experiments [15,34,35].

From EDX analyses, we determined that small amounts of Mg, Ca, P and Cu are present within *L. vulgaris* teeth (see also [46]). Especially the presence of Ca seems to relate to increased values of *E* and *H* (figure 12*d*). This indicates that some cross-linking could be present in these teeth, similar to the vetigastropod *Megathura crenulata* (see [29]) or arthropod cuticle [156–159]. However, potentially, the degree of sclerotization also determines the mechanical properties, similar to the situation in insect cuticle [125,160,161] or in paludomid and nudibranch radular teeth [19,22,23].

### 4.4. Coating of teeth

We found that the tooth regions that have intimate contact with the ingesta possess a coating, which verifies our hypothesis (d) ‘these surfaces show wear-coping mechanisms as coatings'. Even though the radula is constantly secreted in the radular sac by over- and underlain epithelia and is thus renewed [47,162–167], mechanisms for the wear reduction have been previously detected. Wear prevention is well documented for the teeth of Polyplacophora and Patellogastropoda, where very high proportions of Fe and Si are incorporated into the tooth's thick leading edge (i.e. the surface of the tooth that interacts directly with the ingesta) (e.g. [4,8–14,16–18,168,169]). In the almost unmineralized teeth of the nudibranch gastropods *Doris pseudoargus* and *Felimare picta* foraging on Porifera, the cephalaspid gastropod *Gastropteron rubrum*, and some paludomid gastropods, we previously documented a similar mechanism. In these teeth, high proportions of Ca or Si were detected on the interacting surfaces of the teeth [19–23]. However, in comparison with the polyplacophoran and patellogastropod teeth, which are almost completely filled with iron oxide or silica, those teeth only possessed a thin superficial layer. As documented for *D. pseudoargus* and *F. picta*, this thin layer is, however, significantly harder and stiffer (*E*_max_ = 45 GPa and *H*_max_ = 2.3 GPa) than the inner structure of the teeth [23]. For *Loligo vulgaris* teeth, we determined high proportions of Si in the interacting surfaces of the teeth (see figures 9 and 12*d*,*e*). Even though we could not test the mechanical properties of this layer, we propose that it is harder and stiffer than the inner tooth structure, based on the results from the tested nudibranch species: this layer potentially reduces abrasion during interaction.

In the previous studies, we determined that the high inorganic contents of the surface influenced the autofluorescence signal. The teeth of *D. pseudoargus*, which contain high proportions of Ca in their surface, emit a strong signal when excited with the lasers of the lowest wavelengths (the colour blue was attributed to this signal). By contrast, the radulae of *G. rubrum* and *F. picta*, which possessed high Si content in the tooth surfaces, emit a strong autofluorescence signal when excited with the laser of 488 nm wavelength (the colour green was attributed to this signal). In *L. vulgaris* (figure 8), we detect a similar pattern, as the surfaces of the teeth containing higher proportions of Si emit a strong autofluorescence signal with the laser of 488 nm wavelength (here translated to the colour green). This coating could also be identified at fractures in SEM (figure 4*b*). The radular membrane emits a strong autofluorescence with the laser of 405 nm wavelength (here translated to the colour blue) and some with the 555 and 639 nm lasers (red colour). As we could here not detect high content of Ca, we would relate this signal to slightly sclerotized chitin and a high content of proteins, similar to the situation in insect cuticle structures [20,21,124–129]. The tooth bases and parts of the marginal plates showed strong autofluorescence with the 405, 555 and 639 nm lasers (figure 8). We would relate this signal rather to unsclerotized and sclerotized chitin, as no high proportions of inorganics could be determined here, similar to insect cuticle structures.

## Data Availability

All data can be found in the electronic supplementary material [170].
